# Aptamer-Functionalized Gold Nanoparticle Assay for Rapid Visual Detection of Norovirus in Stool Samples

**DOI:** 10.3390/bios15060387

**Published:** 2025-06-16

**Authors:** Maytawan Thanunchai, Sirikwan Sangboonruang, Natthawat Semakul, Kattareeya Kumthip, Niwat Maneekarn, Khajornsak Tragoolpua

**Affiliations:** 1 Division of Clinical Microbiology, Department of Medical Technology, Faculty of Associated Medical Sciences, Chiang Mai University, Chiang Mai 50200, Thailand; sirikwan.sang@cmu.ac.th (S.S.); khajornsak.tr@cmu.ac.th (K.T.); 2 Infectious Diseases Research Unit (IDRU), Faculty of Associated Medical Sciences, Chiang Mai University, Chiang Mai 50200, Thailand; 3 Office of Research Administration, Chiang Mai University, Chiang Mai 50200, Thailand; 4 Department of Chemistry, Faculty of Sciences, Chiang Mai University, Chiang Mai 50200, Thailand; natthawat.semakul@cmu.ac.th; 5 Department of Microbiology, Faculty of Medicine, Chiang Mai University, Chiang Mai 50200, Thailand; kattareeya.k@cmu.ac.th (K.K.); niwat.m@cmu.ac.th (N.M.); 6 Center of Excellence in Emerging and Re-Emerging Diarrheal Viruses, Chiang Mai University, Chiang Mai 50200, Thailand

**Keywords:** Norovirus, gold nanoparticles, aptamer-based assay, colorimetric detection, point-of-care diagnostics

## Abstract

Norovirus (NoV), a leading cause of acute gastroenteritis worldwide, imposes significant morbidity and economic burdens across all age groups. Timely and accurate laboratory diagnosis is crucial for effective outbreak control and patient management. However, current diagnostic methods often require specialized equipment, technical expertise, and considerable time. To address these challenges, we developed a visual detection method utilizing gold nanoparticles (AuNPs) functionalized with the SMV25 aptamer specific to the NoV capsid protein. Detection relies on MgCl_2_-induced changes in the color and absorbance of these aptamer-functionalized AuNPs. The assay exhibited a good linear relationship between the A630/A520 absorbance ratio and NoV capsid protein concentration. Specifically, in a buffer system, this linearity (R^2^ = 0.9026) was observed over a 0–32 ng/µL range with a limit of detection (LOD) of 9.65 ng/µL. Similarly, for NoV spiked into stool suspensions, a strong linear correlation (R^2^ = 0.9170) was found across a 0–100 ng/µL range, with an LOD of 37.11 ng/µL. Evaluation with real stool samples yielded 77% sensitivity and 65% specificity. Notably, the assay demonstrated the highest sensitivity towards NoV GII.2 (100%), followed by GII.4 (78%). Scanning transmission electron microscopy confirmed the underlying aggregation and dispersion patterns of the aptamer-functionalized AuNPs. This colorimetric assay provides a simple, rapid, and visual method for NoV detection. Nevertheless, further enhancements are necessary to improve its performance in the direct testing of complex specimens, paving the way for future on-site detection applications, especially in resource-limited settings.

## 1. Introduction

Norovirus (NoV) is a leading cause of acute gastroenteritis, with millions of cases reported annually worldwide. NoV can spread rapidly through contaminated food, water, surfaces, and person-to-person contact [1]. Given its ability to survive on surfaces for several days and to resist disinfectants, NoV can cause outbreaks, especially in close-contact environments, such as schools, cruise ships, and healthcare facilities. Furthermore, only a few viral particles are required for infection, underscoring the ease of its spread [2]. Exposure to the virus induces vomiting, diarrhea, nausea, and abdominal pain, which lasts 1–3 days. Although most cases are mild, dehydration can be a significant risk factor for disease severity, particularly for young children, older adults, and immunocompromised individuals. NoV genogroup II (GII) is the most common genogroup linked to human infections, with the GII.4 genotype being the major cause of outbreaks and sporadic cases worldwide [1,3].

Accurate diagnosis of NoV infection is crucial for effective outbreak management and epidemiological tracking. NoV cannot be cultivated in conventional cell culture systems; thus, molecular and immunological methods are required to detect genetic material or viral antigen in clinical samples for laboratory diagnosis. Reverse transcription polymerase chain reaction (RT-PCR) is the gold standard for diagnosing NoV. It detects and quantifies viral RNA with high sensitivity and specificity, often providing differentiation between genogroup and strains [4,5]. However, RT-PCR has certain limitations that may reduce its practicality in low-resource settings, as it requires specialized equipment, specific reagents, and trained personnel. Moreover, the process involves multiple steps, including RNA extraction, reverse transcription, amplification, and analysis, which can be time-consuming. In contrast, immunological methods have lower sensitivity and specificity than molecular techniques. These methods often fail to detect low viral loads, resulting in an increased risk of false-negative results, particularly in early-stage or asymptomatic infections. Furthermore, the antigenic diversity of norovirus strains can compromise the binding efficiency of the antibodies used in the immunoassays [6,7]. The limitations of RT-PCR and immunological methods make them impractical in urgent diagnostic situations. Therefore, alternative, rapid, simplified, and cost-effective testing methods are needed for screening, identifying outbreaks, and supporting epidemiological studies.

Gold nanoparticles (AuNPs) are a versatile tool in rapid diagnostic technologies because of their unique physical, chemical, and optical properties. Specifically, their unique optical properties are attributed to surface plasmon resonance (SPR), a phenomenon in which free electrons on the nanoparticle surface resonate with incident light at specific wavelengths. The SPR characteristics of AuNPs vary with their size and shape. Spherical AuNPs generally exhibit a single SPR peak within the visible spectrum. Smaller AuNPs (5–20 nm) typically appear red because of their absorption properties, whereas larger particles tend to scatter light more and show color shifts tending toward purple (redshift) [8,9,10]. AuNPs provide a high surface area for the immobilization of diagnostic probes, such as antibodies, aptamers, and nucleic acids, which amplify signal detection [11,12,13].

Aptamers are short synthetic nucleic acid sequences that bind to specific molecular targets with high affinity and specificity. They are preferable to antibodies because of their greater stability, ease of synthesis, and the ability to target a wide range of analytes, including proteins, small molecules, and whole viruses [14]. The functionalization of AuNPs with aptamers facilitates rapid diagnostic testing, which often produces simple colorimetric outputs that are visible to the naked eye, making them promising alternative probes for diagnostic assays [15,16,17]. Aptamer-functionalized AuNPs have been developed for the colorimetric detection of various pathogens, such as SARS-CoV-2 [18], Zika virus [19], and *Pseudomonas aeruginosa* [20], using aptamers specific to pathogen-associated proteins. Furthermore, this detection system is applied in targeting specific cancer biomarkers, hormones, or small molecules in clinical samples with high precision [11,21,22].

SMV25 is a single-stranded DNA aptamer that targets the NoV capsid protein [23]. It was developed through the systematic evolution of ligands by exponential enrichment (SELEX) using Snow Mountain virus (SMV), the prototype GII.2 human norovirus (HuNoV), as the selection target. Secondary structural prediction revealed a dominant loop and two protruding hairpins (Figure S1). The binding capacity of SMV25 was evaluated against various NoV-like particles (VLPs) and HuNoV in outbreak-derived stool specimens. Its signal intensity ratio (T/N ratio) indicated that SMV25 exhibited a strong binding affinity for GII.2 and GII.4, followed by GII.1, GII.7, GII.6, GI.8, and GII.3 VLPs. Similarly, SMV25 displayed high T/N ratios for GII.2 (SMV), GII.4, and GII.1 in diluted, partially purified stool samples from outbreaks. Furthermore, SMV25 demonstrated high capture efficiency during virus preconcentration before performing RT-PCR analysis of artificially contaminated lettuce samples. To date, the SMV aptamer has not been exploited in combination with AuNPs as a biosensing material for the development of simple colorimetric assays for NoV detection.

**Figure 1:**
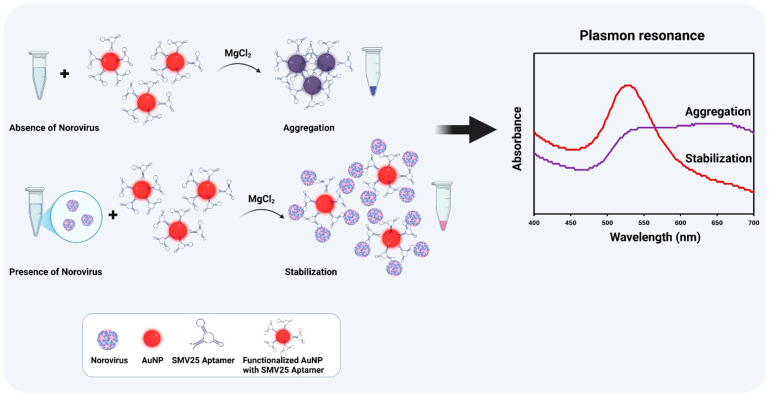
A schematic illustration of the Snow Mountain virus (SMV25) aptamer-functionalized gold nanoparticle (AuNP)-based colorimetric assay for norovirus detection.

Addressing this gap and highlighting a significant step forward in NoV diagnostics, this study introduces an innovative approach: we have developed and validated a simple, user-friendly, and cost-effective colorimetric method for the direct detection of NoV in stool samples by employing SMV25 aptamer-functionalized AuNPs. The novelty of our assay lies not only in this first-time combination but also in its specific design for direct application to complex matrices and its operational simplicity. Detection is based on the visible red color obtained in the reaction following MgCl_2_ induction after aptamer–NoV binding. The red color arises from enhanced steric stabilization, which reduces AuNP aggregation and minimizes the wavelength shift in the surface plasmon absorbance. In contrast, in the absence of NoV, the net charge of the functionalized AuNPs decreases due to neutralization by magnesium ions (Mg^2+^), promoting particle aggregation and causing a redshift in the surface plasmon absorbance toward higher wavelengths. In addition to visible colorimetric detection, the absorbance spectra were measured between 400 and 700 nm using spectral scanning spectrophotometers (Figure 1). The absorbance ratio at 630 nm to 520 nm indicates the aggregation levels; a higher ratio signifies greater aggregation (with increased absorbance at 630 nm), whereas a lower ratio indicates dispersed nanoparticles, the absorbance of which is stronger at 520 nm. The significance of this work is underscored by its potential as a screening tool; this functionalized AuNP-based colorimetric assay provides crucial proof-of-concept for detecting NoV without requiring specialized equipment or highly trained personnel. This makes the assay highly useful for rapid, on-site diagnostics in resource-limited environments, thereby facilitating timely outbreak response and improving public health surveillance capabilities for norovirus.

## 2. Materials and Methods

### 2.1. Aptamer

The SMV25 aptamer (5′-AGTATACGTATTACCTGCAGCCATCTGTGTGAAGACTATATGGCGCTCACATATTTCTTTCCGATATCTCGGAGATCTTGC-3′) [23] was obtained from Bio Basic Inc., Markham, ON, Canada. The aptamer was modified with thiols at its 3′ end using high-performance liquid chromatography for purification and verification. The thiolated end group would facilitate the attachment of the aptamer onto the AuNP surfaces. Prior to immobilization, the disulfide bonds in the thiolated end of the aptamer were reduced by treatment with Tris (2-carboxyethyl) phosphine hydrochloride (TCEP, Sigma Aldrich, Oakville, ON, Canada) at a molar ratio of 10:1 (TCEP:aptamer), following the manufacturer’s recommendations. Briefly, 10 μM of aptamer solution was incubated with 0.1 mM TCEP for 1 h in the dark. The reduced aptamer required no additional purification or dialysis because TCEP does not interfere with thiol–gold binding. To restore the active conformation of the aptamer, it was refolded by heating at 85 °C for 5 min in a water bath, followed by rapid cooling in an ice bath.

### 2.2. The Synthesis and Characterization of the AuNPs

Citrate-capped AuNPs were synthesized by sodium citrate reduction of HAuCl_4_, as described by Zhang et al. [24], with some modifications. Briefly, an aqueous solution of 1 mM HAuCl_4_ (100 mL) (Sigma-Aldrich, St. Louis, MO, USA) was reduced with a 38.8 mM trisodium citrate solution (10 mL) (RCI Labscan Ltd., Bangkok, Thailand) at 90 °C under vigorous stirring for 15 min, resulting in a color change in the mixture from pale yellow to a reddish wine-like color, indicating nanoparticle formation. The solution was boiled, stirred for 10 min, and cooled to room temperature (RT). The solution was then filtered through a 0.45-mm membrane filter and stored in dark glass bottles at 4 °C. The particle size and zeta potential (ZP) of the AuNPs were measured by dynamic light scattering with a Zetasizer (Malvern Instruments, Worcestershire, UK). The AuNPs were then visualized by scanning transmission electron microscopy (STEM). A 10 μL portion of the sample was dropped onto a piece of a carbon-coated copper grid and allowed to air-dry. STEM images were acquired using a JSM-IT800 Ultrahigh Resolution Field Emission SEM (JEOL, Peabody, MA, USA). UV–visible (UV–Vis) spectroscopy was acquired, and spectral analysis was performed at wavelengths between 400 and 700 nm.

### 2.3. The Immobilization of the SMV25 Aptamer onto AuNPs

The SMV25 aptamer was immobilized onto AuNPs, first by preparing 2.5 μM of the SMV25 aptamer by dilution of a 10 μM stock in phosphate-buffered saline (PBS; HyClone, Logan, UT, USA) containing 0.55 mM MgCl_2_ (Sigma Aldrich, Canada). Subsequently, 5 μL of the 2.5 μM aptamer solution was combined with 25 μL of AuNPs and incubated at RT for 16 h. This process utilized the strong affinity of the thiol groups for the gold surfaces to establish the direct immobilization of the thiolated aptamer onto the AuNPs by adsorption.

### 2.4. Optimization of MgCl_2_ Concentration for SMV25-Functionalized AuNP Aggregation

To develop the colorimetric detection method, the effect of MgCl_2_ concentration on the aggregation of AuNPs was investigated. A mixture was prepared by combining 30 μL AuNPs, 5 μL PBS, and 20 μL MgCl_2_ at varying concentrations (8, 10, 12, 14, 16, and 18 mM) in a 0.2 mL PCR tube, yielding a total volume of 55 μL. The color change was observed with the naked eye immediately after the addition of MgCl_2_; the reddish color indicates dispersed AuNPs, which was interpreted as positive, while the purple indicates aggregated AuNPs, which was interpreted as negative. Furthermore, absorbance intensity changes were recorded using a UV–Vis spectrophotometer (BioTek, Agilent, Winooski, VT, USA) within the wavelength range of 400–700 nm. The absorbance ratio at 630 nm to 520 nm (A630/520) was analyzed. An A630/520 value ≥ 1 indicated aggregation, which was interpreted as a negative result, whereas A630/520 < 1 indicated dispersed AuNPs, which was interpreted as a positive result.

### 2.5. The Detection of the NoV Capsid Protein

The recombinant NoV group-2 capsid protein was obtained from ProSpec, Rehovot, Israel. Various concentrations (0, 2, 4, 8, 16, and 32 ng/μL) of the protein were prepared by dilution in PBS, after which 5 μL of each concentration was mixed with 30 μL of SMV25-modified AuNP solution in PCR tubes. The mixtures were incubated at RT for 30 min. Following incubation, 20 μL of 14 mM MgCl_2_ was added to each sample. The resulting color changes were visually inspected. As mentioned above, a red solution indicates a positive result, whereas a purple solution indicates a negative result. The absorbance intensities were measured using a UV–visible spectrophotometer at the 400–700 nm wavelength. The absorbance ratio at 630 nm to 520 nm (A630/520) was calculated and interpreted as above.

For the spiked assay, a 10% stool suspension was prepared from a healthy donor by weighing 1 g of stool and homogenizing it in 9 mL of PBS (pH 7.4) using a vortex mixer. The suspension was then aliquoted and stored at −20 °C until use. Prior to testing, the NoV capsid protein was spiked into the 10% stool suspension at final concentrations of 12.5, 25, 50, and 100 ng/μL. Subsequently, 5 μL of each spiked sample was added to 30 μL of SMV25-modified AuNP solution and incubated at RT for 30 min. As described in the abovementioned protocol, 20 μL of 14 mM MgCl_2_ was added to the mixture, and changes in color and absorbance intensities were recorded and analyzed.

### 2.6. Detection of NoV in Stool Samples

Ninety stool samples from patients with diarrhea were kindly provided by Professor Niwat Maneekarn of the Center of Excellence (Emerging and Re-emerging Diarrheal Viruses), Chiang Mai University. All samples had been previously tested by real-time RT-PCR, with the genogroup and genotypes determined for the positive cases.

For analysis, 5 μL of each stool suspension was directly added to the SMV25-functionalized AuNP solution. In cases where the samples appeared turbid or dark brown, the suspensions were diluted 1:1 with PBS before being mixed with the AuNPs. The mixtures were incubated at RT for 30 min, followed by the addition of 20 μL of 14 mM MgCl_2_. The color changes were observed visually, and the absorbance intensities were measured. Quantitative assessment was performed using the calibration equation derived from the NoV capsid protein spiked stool suspension experiment. Finally, the assay’s sensitivity and specificity were analyzed using the MedCalc diagnostic test evaluation version 23.2.6, an online statistical calculator [25].

### 2.7. The STEM of the SMV25-Functionalized AuNPs

STEM was performed to visualize the aggregation and dispersion patterns of various reactions involving the SMV25-functionalized AuNPs. The test conditions were as follows: (1) SMV25-functionalized AuNPs alone; (2) SMV25-functionalized AuNPs in the presence of 32 ng/µL of NoV capsid protein, representing a positive reaction; (3) SMV25-functionalized AuNPs with PBS as the negative control; (4) SMV25-functionalized AuNPs with a NoV-positive stool sample; and (5) SMV25-functionalized AuNPs with a NoV-negative stool sample. Following the addition of 14 mM MgCl_2_ and the observation of the resulting colorimetric reaction, a 10 µL portion of each sample was placed onto a carbon-coated copper grid and air-dried. STEM images were then captured using the JSM-IT800 Ultrahigh Resolution Field Emission SEM (JEOL, Tokyo, Japan).

## 3. Results

### 3.1. The Characterization of the AuNPs

The AuNPs were successfully synthesized with a particle size of approximately 20.52 ± 1.16 nm. Citrate capping conferred a negative surface charge on the AuNPs with a ZP value of −36.02 ± 1.11 mV. The morphological characteristics and monodispersed particles were visualized by STEM. The UV–vis spectra of the AuNPs showed a major peak at 520 nm, which confirmed that the particle size of the AuNPs was within 15–20 nm (Figure S2). Based on their particle size (20.52 nm diameter via DLS; ~13.12 nm via STEM) and an optical density (OD) of 0.8, the weight concentration of the synthesized AuNPs was estimated to be 0.0382–0.0387 mg/mL.

### 3.2. The Effect of MgCl_2_ on the Aggregation of SMV25-Functionalized AuNPs

**Figure 2:**
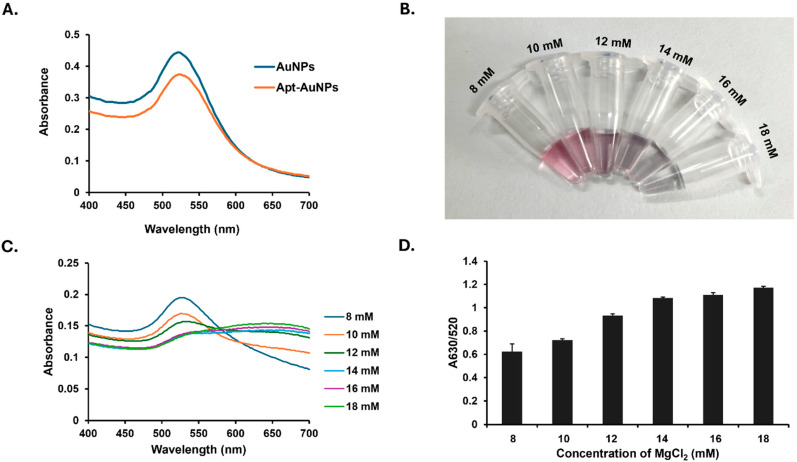
The functionalization of gold nanoparticles (AuNPs) with a Snow Mountain virus (SMV25) aptamer specific to the NoV capsid protein and the effect of MgCl_2_ on the aggregation of the functionalized AuNPs. (**A**) UV–visible spectra comparing unmodified AuNPs and SMV25 aptamer-functionalized AuNPs. (**B**) The aggregation behavior of the functionalized AuNPs in response to increasing MgCl_2_ concentration (8–18 mM). A visible color change from reddish to purple appeared at 14 mM MgCl_2_ and above. The reddish color indicates the stability of the functionalized AuNPs, while purple indicates AuNP aggregation with MgCl_2_ addition. (**C**) The UV–visible absorbance spectra of the functionalized AuNP suspensions with different MgCl_2_ concentrations, demonstrating a shift in the absorbance peak at 14 mM MgCl_2_ and higher. (**D**) Changes in the absorbance ratio with increasing MgCl_2_ concentration.

To develop aptamer-functionalized AuNPs, the SMV25 aptamer was immobilized onto the AuNP surface via thiol–gold interactions. The SMV25-functionalized AuNPs retained the same absorbance peak at 520 nm as the unmodified AuNPs, indicating that conjugation did not alter their optical density (Figure 2A). The effect of MgCl_2_ concentration on the aggregation of the functionalized AuNPs was then evaluated. Unmodified AuNPs became destabilized at MgCl_2_ concentrations of 1 mM or higher, as indicated by a visible color change from reddish to purple-gray and A630/A520 > 1 (Figure S3). However, the SMV25-functionalized AuNPs displayed greater stability and required a higher MgCl_2_ concentration to undergo aggregation. This result indicates that the aptamers inhibit the interaction between the AuNP surface charges and MgCl_2_ counterions. Figure 2B shows a progressive color change from reddish to purple with increasing MgCl_2_ concentration. Furthermore, the absorbance peak exhibited a redshift from 520 nm to 630 nm with increasing MgCl_2_ concentration (Figure 2C), causing a gradual increase in the aggregation-induced A630/A520 ratio (Figure 2D), with optimal aggregation achieved at 14 mM MgCl_2_ (A630/A520 > 1). Thus, the 14 mM MgCl_2_ concentration was applied in subsequent experiments.

### 3.3. The Detection of the NoV Capsid Protein Using SMV25-Functionalized AuNPs

**Figure 3:**
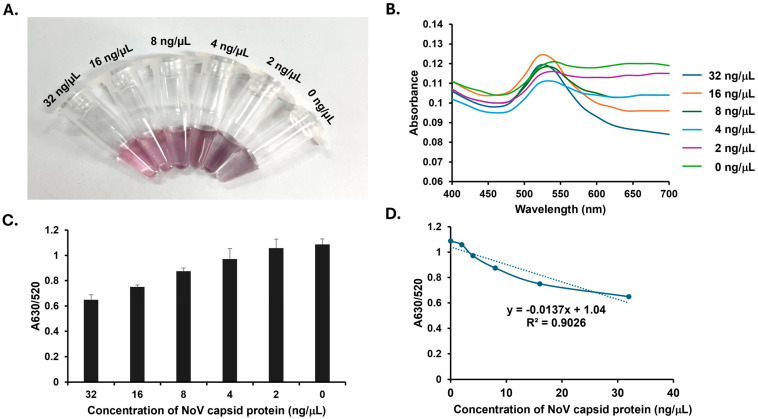
Norovirus (NoV) capsid protein detection using Snow Mountain virus (SMV25) aptamer-functionalized gold nanoparticles (AuNPs). (**A**) The aggregation of SMV25-functionalized AuNPs in response to varying concentrations of the recombinant NoV capsid protein (0–32 ng/μL). (**B**) UV–visible spectra showing the absorbance profiles of AuNPs at different NoV capsid protein concentrations. (**C**) The absorbance ratio (A630/A520) indicating dose-dependent aggregation. (**D**) A calibration curve plotting the A630/A520 ratio against varying concentrations of the NoV capsid protein (0–32 ng/µL).

After optimizing the MgCl_2_ concentration, the detection of the NoV capsid protein was performed. The addition of SMV25-functionalized AuNPs elicited a distinct, concentration-dependent response. With decreasing capsid protein concentration, the reaction’s color progressively shifted from red to purple. This visual change indicates that at higher protein concentrations, the binding between the aptamer and target protein stabilizes the AuNPs, thereby minimizing aggregation. Conversely, the aggregation of AuNPs occurred when the capsid protein was absent or at low concentrations (Figure 3A). This behavior was corroborated by UV–vis spectroscopy, which revealed a peak absorbance shift from 520 nm to 630 nm as aggregation increased (Figure 3B). Furthermore, the A630/A520 ratio increased as the recombinant protein concentration decreased (Figure 3C), consistent with the observed color transition. The calibration curve, plotting the A630/A520 ratio against the NoV capsid protein concentration, is presented in Figure 3D. This curve clearly demonstrates a good linear relationship (R^2^ = 0.9026) between the A630/A520 values and NoV capsid protein concentrations over the range of 0 to 32 ng/µL. Using the formula LOD = (3 × standard deviation of blank sample)/slope of the calibration curve, the LOD was calculated to be 9.65 ng/µL.

**Figure 4:**
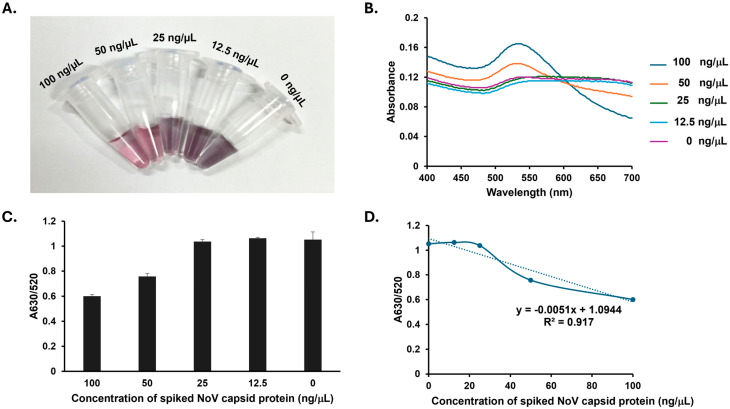
The detection of the spiked norovirus (NoV) capsid protein in stool suspension using Snow Mountain virus (SMV25) aptamer-functionalized gold nanoparticles (AuNPs). (**A**) Changes in colorimetric detection under various NoV capsid protein concentrations (0, 12.5, 25, 50, and 100 ng/μL) in the stool samples. (**B**) The UV–visible spectra of SMV25-functionalized AuNPs detecting the NoV capsid protein spiked into the stool suspension. (**C**) The absorbance ratio (A630/A520) indicating dose-dependent aggregation. (**D**) A calibration curve plotting the A630/A520 ratio against varying concentrations of NoV capsid protein (0–100 ng/µL).

To evaluate the method’s performance in complex biological samples, varying concentrations of recombinant NoV capsid protein (0, 12.5, 25, 50, and 100 ng/µL) were spiked into stool suspensions from healthy donors, simulating diarrheal conditions. Visually, the capsid protein was effectively detected at concentrations of 50 and 100 ng/µL, where the solution color indicated AuNP stability. Below 50 ng/µL, however, the reaction turned purple due to AuNP aggregation. For quantitative assessment, a calibration curve was plotted using the A630/A520 ratio against the NoV capsid protein concentration spiked in the stool suspension (Figure 4D). This curve clearly demonstrates a good linear relationship (R^2^ = 0.9170) between the A630/A520 values and the spiked protein concentrations across the 0 to 100 ng/µL range. From these data, the LOD was calculated to be 37.11 ng/µL. Additionally, the inter-assay reproducibility of our method was evaluated by calculating the coefficient of variation (%CV). Our findings indicated %CV values ranging from 2.24% to 8.42% for the colorimetric detection of the NoV capsid protein in a buffer system and from 0.70% to 5.99% for the detection of spiked NoV in stool suspension. These %CVs, determined from three independent experiments, affirm the strong inter-assay precision of our method. Such robust reproducibility highlights the capability of the SMV25-functionalized AuNPs for the consistent and reliable detection of NoV capsid proteins.

### 3.4. Evaluating the Sensitivity and Specificity of SMV25-Functionalized AuNPs

To assess the effectiveness of SMV25-functionalized AuNPs, 90 stool samples were analyzed using the AuNP aptamer detection system. Of these, 70 had previously been confirmed as positive, whereas 20 had been confirmed as negative for NoV by real-time RT-PCR. Our method achieved a sensitivity of 77% and specificity of 65% (Table 1), and the functionalized AuNPs successfully detected multiple genotypes. The highest positivity rate was recorded for genotype GII.2 (100%), followed by GII.4 (78%). The sensitivity for other genotypes, such as GII.17 and GII.3, was slightly lower at 66.7% and 62.5%, respectively (Table 2). For stool samples that tested negative for NoV but contained other viral gastroenteritis pathogens, such as rotavirus, sapovirus, adenovirus, enterovirus, and astrovirus, the SMV25-functionalized AuNPs demonstrated 100% specificity in rotavirus- and sapovirus-containing samples. Of the astrovirus-containing samples, 1 in 3 tested positive (66.7%), while the lowest specificity was observed in the adenovirus and enterovirus samples (33.3%; Table 3).

### 3.5. Scanning Transmission Electron Microscopy Analysis

**Figure 5:**
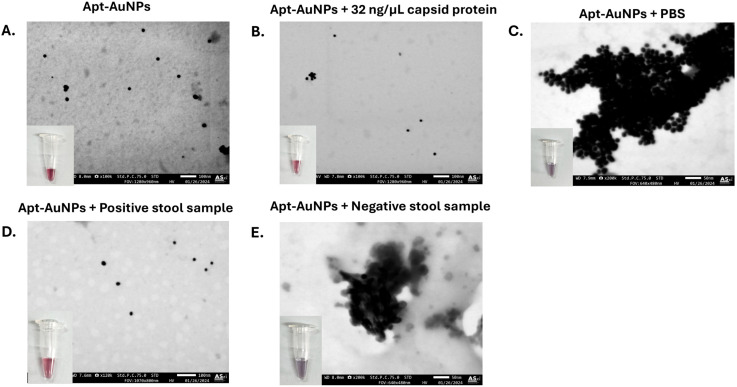
Scanning transmission electron microscopy (STEM) analysis of Snow Mountain virus (SMV25) aptamer-functionalized gold nanoparticles (AuNPs) under various conditions. STEM images of SMV25-functionalized AuNPs under different experimental conditions, showing variations in morphology and aggregation patterns: (**A**) SMV25-functionalized AuNPs; (**B**) functionalized AuNPs incubated with 32 ng/μL NoV capsid protein; (**C**) SMV25-functionalized AuNPs treated with phosphate-buffered saline; (**D**) SMV25-functionalized AuNPs with stool samples containing NoV; (**E**) SMV25-functionalized AuNPs with negative stool samples. The aggregation and dispersion behaviors of the AuNPs under each condition were observed following the addition of 14 mM MgCl_2_.

The dispersion and aggregation behaviors of the functionalized AuNPs under various conditions were analyzed by STEM. As mentioned earlier, the immobilization of the aptamer on the gold surface did not affect the optical density of the nanoparticles. This was confirmed on STEM images, showing that the functionalized AuNPs retained a similar size and dispersion pattern as their unmodified counterparts (Figure 5A). The AuNPs remained dispersed at 32 ng/µL NoV capsid protein concentration (Figure 5B), whereas, at 0 ng/µL, they exhibited aggregation (Figure 5C). Furthermore, dispersion and aggregation patterns observed in both positive and negative stool samples were consistent with the colorimetric results, as shown in Figure 5D,E, respectively.

## 4. Discussion

Several biosensor-based approaches have recently been developed as alternative methods for NoV detection to address the limitations of existing techniques. RT-PCR, the gold standard, while highly sensitive, is costly and time-consuming. Furthermore, immunological assays may fail to detect emerging NoV genotypes with high sensitivity. AuNP-based biosensors offer a promising alternative detection system that is portable and efficient. Despite these advantages, additional optimization is required to adapt these methods for routine use, including outbreak monitoring and public health surveillance.

This study explored the potential of aptamer-functionalized AuNPs as an innovative, rapid, and accessible platform for detecting NoV capsid proteins. The aptamer was modified by adding a thiol (-SH) group at the 3′ end, enabling its immobilization onto the AuNP surface via thiol–gold interactions. Thiolated aptamers bind to AuNPs by covalent bonding, ensuring strong and highly stable immobilization. This bonding enhances target recognition by reducing steric hindrance, thereby facilitating high sensitivity and long-term diagnostic assays. In contrast, non-thiolated aptamers offer a simpler and more cost-effective alternative but may exhibit reduced stability and reproducibility [26,27]. The salt-induced aggregation in the assay generated a detectable signal, manifesting as a shift in localized SPR visible by naked-eye visualization. NaCl and MgCl_2_ are commonly used to induce the aggregation of AuNPs in colorimetric assays. The choice between the two depends on the specific biosensing application; NaCl is preferred for gradual and tunable aggregation, while MgCl_2_ is favored for rapid and strong aggregation at lower concentrations [28,29]. In this study, strong aggregation was necessary for detecting the NoV capsid protein in stool samples. Therefore, MgCl_2_, a divalent salt, was used as a coagulant because of its superior ability to neutralize surface charges, thereby inducing the robust aggregation of aptamer-functionalized AuNPs. Typically, AuNPs are stabilized in solution by a negative surface charge from the capping citrate ions. These charges create repulsive forces that prevent the nanoparticles from aggregating. When MgCl_2_ is introduced, Mg^2+^ neutralizes this surface charge by binding to negatively charged groups, weakening the electrostatic repulsion, thereby inducing aggregation. Similar studies have demonstrated the capability of nanoparticle systems for colorimetric detection. Aithal et al. [18] used MgCl_2_ to induce nanoparticle aggregation for SARS-CoV-2 spike protein detection, achieving sensitivities as low as 16 nM of spike protein in PBS and 3540 genome copies/μL of inactivated SARS-CoV-2 virus. Djisalov et al. [30] demonstrated the rapid detection of *Trichoderma* spp. via the MgCl_2_-induced aggregation of AuNPs, which was followed by color change evaluation and absorbance spectra measurement. These examples highlight the potential of simple, equipment-free diagnostic testing using visual or absorbance measurements.

Our study successfully demonstrated the detection of NoV capsid proteins in both colloidal and stool suspensions using aptamer–AuNP conjugation, as confirmed by naked-eye visualization and absorbance measurements. The method exhibited a consistent response in both matrices, with A630/A520 values showing a clear linear increase as the NoV capsid protein concentration decreased. Specifically, for the colloidal suspension, this linear relationship was observed over the 0–32 ng/µL range, yielding a regression correlation coefficient (R^2^) of 0.9026. In the stool suspension, the linear trend extended across the 0–100 ng/µL range, with an R^2^ value of 0.9170. These strong correlations, both approaching to 1, underscore the consistency and reliable response of the method, indicating its suitability for sensitive norovirus detection. STEM further confirmed the reliability of the system by demonstrating the well-defined dispersion and aggregation patterns, which are consistent with UV–vis spectroscopy findings. However, in real stool samples, the detection efficiency of our assay decreased, yielding 77% sensitivity and 65% specificity. This reduction can be primarily attributed to the highly complex and heterogeneous nature of stools. As a biological matrix, stool contains bacteria, undigested food, metabolites, and other diverse components that can interfere with the assay in multiple ways [31]. For example, physical interference can arise from high turbidity or strong coloration in some samples, which may obscure the colorimetric readout. Additionally, various components within the stool matrix can engage in non-specific interactions with either the aptamers or the AuNPs, leading to unintended aggregation or stabilization events. The biochemical complexity of stool further impairs specific probe–target interactions; key contributing factors include potential enzymatic degradation of aptamers or the NoV capsid protein target, which diminishes available binding components, and variations in pH and ionic strength that can adversely alter AuNPs, aptamer conformation, and binding affinity. Collectively, these matrix-inherent factors can obscure signals and reduce overall assay performance [32,33]. Beyond these direct matrix effects, pre-analytical variables associated with sample handling and storage also significantly influence real-world reproducibility [34]. Repeated freeze–thaw cycles, for example, can degrade target analytes, and inconsistencies in dilution protocols may introduce variability in the final analyte concentrations presented to the assay [35,36]. In our experiments, certain 10% stool suspension samples were further diluted 1:1 with PBS if they exhibited high turbidity or coloration that interfered with colorimetric detection. This pre-treatment proved critical for some highly turbid, RT-PCR-positive samples. Specifically, the dilution step resulted in a lower A630/A520 ratio compared to their undiluted counterparts, which enables a correct positive interpretation where the undiluted sample might have yielded false-negative results. These corrected interpretations were consistent with RT-PCR findings. This observation underscores the trade-off; while dilution effectively reduces matrix-induced interference, it unavoidably lowers the absolute concentration of viral particles, which can, in turn, decrease detection sensitivity. To address challenges stemming from both inherent matrix effects and sample management issues, future investigations could explore strategies such as optimizing pH and ionic strengths, establishing standardized sample handling/preparation procedures, and preparing matrix-matched calibrations to enhance detection robustness in complex samples. Furthermore, conducting a study to determine an optimal, standardized dilution factor by quantitatively assessing its impact on sensitivity would be valuable in refining the assay for routine field use.

Notably, the system detected multiple NoV genotypes with high sensitivity, particularly for GII.2 (100%) and GII.4 (78%). This result is largely due to the use of NoV GII.2 as the target for aptamer selection. The binding capacity of the selected aptamer to various VLPs had been previously investigated, and strong binding affinity was observed for GII.2 and GII.4, followed by GII.6 and GII.7, with slightly lower affinity for GII.3 and GII.17 [23]. These results were consistent with our findings. During specificity testing in the present study, the assay showed no false-positive results in samples containing rotavirus or sapovirus, but did produce false positives for samples containing astrovirus, adenovirus, and enterovirus. However, definitive conclusions about the cross-reactivity with these viruses could not be made. Further validation using intact viruses or recombinant viral proteins is necessary to confirm the specificity of the assay for other gastroenteritis-causing viruses. Escudero-Abarca et al. [23] reported that SMV25 exhibited significantly low T/N ratios for poliovirus and hepatitis A virus from cell culture lysates, indicating that it may not bind to non-target viruses. The false-positive results observed in the present study may have been caused by interfering substances in the stools that destabilized the AuNPs.

Various aptamer- and AuNP-based biosensors have been developed for NoV detection using fluorescence and electrochemical systems. Giamberardino et al. [37] designed a biosensor that integrated a DNA aptamer with an AuNP-modified screen-printed carbon electrode to detect both HuNoV and murine norovirus in stool and environmental specimens using fluorescence anisotropy. Weng and Neethirajan [38] developed a portable fluorometric paper-based microfluidic device that combined aptamers to detect norovirus spiked into food matrices. Similarly, Li et al. [39] introduced a dual-mode aptasensor incorporating AuNPs that enabled both electrochemical and colorimetric NoV detection in fecal samples. Although these methods offered high sensitivity, they often involved complex procedures and required expensive instrumentation. Our approach used a simple mix-and-detect format, which could reduce the operational difficulty and costs of detection. However, the main limitation of this study is that we did not use live NoV or other gastroenteritis viruses to assess assay performance. The amount of recombinant capsid protein may not accurately reflect the actual virus levels in samples.

In summary, SMV25-functionalized AuNPs present a promising approach for NoV detection. The assay demonstrated high sensitivity in the buffer systems, although its performance in complex biological samples requires further optimization. Future research should focus on improving specificity and ensuring consistent applicability across genotypes to enhance practical implementation.

## 5. Conclusions

This study successfully developed SMV25-functionalized AuNPs demonstrating high specificity for the NoV capsid protein. The detection mechanism relied on color changes and absorbance peak shifts resulting from AuNP aggregation. The calculated LOD for the NoV capsid protein was 9.65 ng/µL in PBS, which increased to 37.11 ng/µL in spiked stool suspension. This difference highlights that interfering substances within the complex stool matrix affected detection performance. Validation with real-world clinical samples yielded an overall sensitivity of 77% and a specificity of 65%. Among the genotypes tested, GII.2 was detected with the highest sensitivity (100%), followed by GII.4 (78%). This colorimetric assay provides a simple, rapid, and visually evaluable method for NoV detection. However, further optimization is crucial to enhance its performance in complex specimens, thereby improving its effectiveness for on-site applications, particularly in resource-limited settings.

## Figures and Tables

**Table 1:** Sensitivity and specificity of the SMV25-functionalized AuNPs.

		SMV-Functionalized AuNPs		Total (n)	Sensitivity/Specificity (%)
		Positive	Negative		
**Real-time PCR**	**Positive**	54	16	70	77
	**Negative**	7	13	20	65

SMV, Snow Mountain virus aptamer; AuNPs, gold nanoparticles; PCR, polymerase chain reaction.

**Table 2:** Positivity rates for different NoV genotypes.

Genotypes	Positive	Negative	Total	% Positive
GII.2	4	0	4	100
GII.3	5	3	8	62.5
GII.4	39	11	50	78
GII.6	1	0	1	100
GII.7	1	0	1	100
GII.17	4	2	6	66.7
	54	16	70	

NoV, norovirus.

**Table 3:** Specificity of SMV25-functionalized AuNPs on other gastroenteritis-causing viruses.

	Positive	Negative	Total	% Negative
Rotavirus	0	3	3	100
Sapovirus	0	3	3	100
Astrovirus	1	2	3	66.7
Adenovirus	2	1	3	33.3
Enterovirus	2	1	3	33.3

SMV25, Snow Mountain virus aptamer; AuNPs, gold nanoparticles.

## Data Availability

The data that support the findings of this study are available from the corresponding authors upon request.

## Supplementary Materials

The following supporting information can be downloaded at: https://www.mdpi.com/article/10.3390/bios15060387/s1, Figure S1: The secondary structure of the SMV-25 aptamer. The structure and related information were obtained from Aptagen (https://www.aptagen.com/aptamers/human-norovirus-smv-25/ accessed on 6 April 2025) and Escudero-Abarca, B. I. et al. [1] (cited in the text as [23]). The red box highlights a common motif shared with other SMV aptamers reported in the study, suggesting a conserved interaction site with HuNoV. The blue and red dots represent A–T and C–G base pairs, respectively. Figure S2: Gold nanoparticle (AuNP) synthesis and characterization. Characterization of the AuNPs by size distribution (A), zeta potential measurements (B), STEM image (C), and spectral analysis at the wavelength between 400 and 700 nm (D), confirming successful synthesis. Figure S3: The effect of MgCl_2_ on the aggregation of unmodified AuNPs. Different concentrations of MgCl_2_ were added to unmodified AuNPs, and the color changes, absorbance spectra, and the A630/520 absorbance ratio were analyzed. (A) The aggregation of unmodified AuNPs in response to increasing MgCl_2_ concentrations (1, 2, 4, 6, 8, 10 mM). A visible color change from reddish to purple-gray started at 1 mM MgCl_2_ or above. (B) The UV–visible absorbance spectra of unmodified AuNPs suspensions with different MgCl2 concentrations, demonstrating a redshift at 1 mM MgCl_2_ or higher. (C) The increase in absorbance ratio (A630/520) following MgCl_2_ addition.
