# Supplementary material for: Aptamer-Functionalized Gold Nanoparticle Assay for Rapid Visual Detection of Norovirus in Stool Samples
Source: Biosensors (Basel). 2025 Jun 16;15(6):387. doi: 10.3390/bios15060387 (PMC12190990; doi:10.3390/bios15060387)
Supplement: Supplementary file 1 [file biosensors-15-00387-s001.zip › biosensors-3668599-supplementary.pdf]

Supplementary information

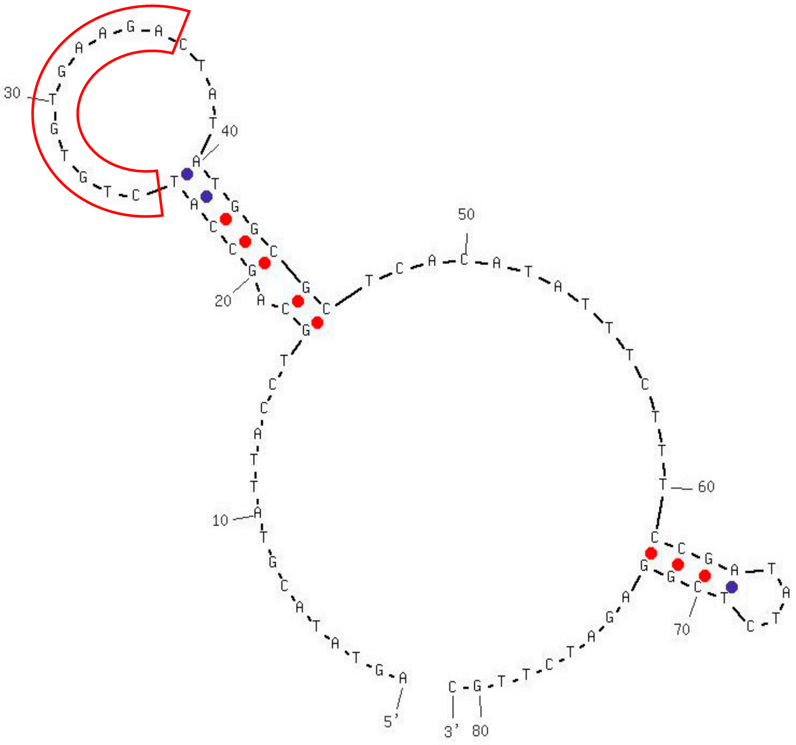

Figure S1: The secondary structure of the SMV-25 aptamer. The structure and related information were obtained from Aptagen (https://www.aptagen.com/aptamers/human-norovirus-smv-25/ accessed on 6 April 2025) and Escudero-Abarca, B. I. et al. [1] (cited in the text as [23]). The red box highlights a common motif shared with other SMV aptamers reported in the study, suggesting a conserved interaction site with HuNoV. The blue and red dots represent A–T and C–G base pairs, respectively.

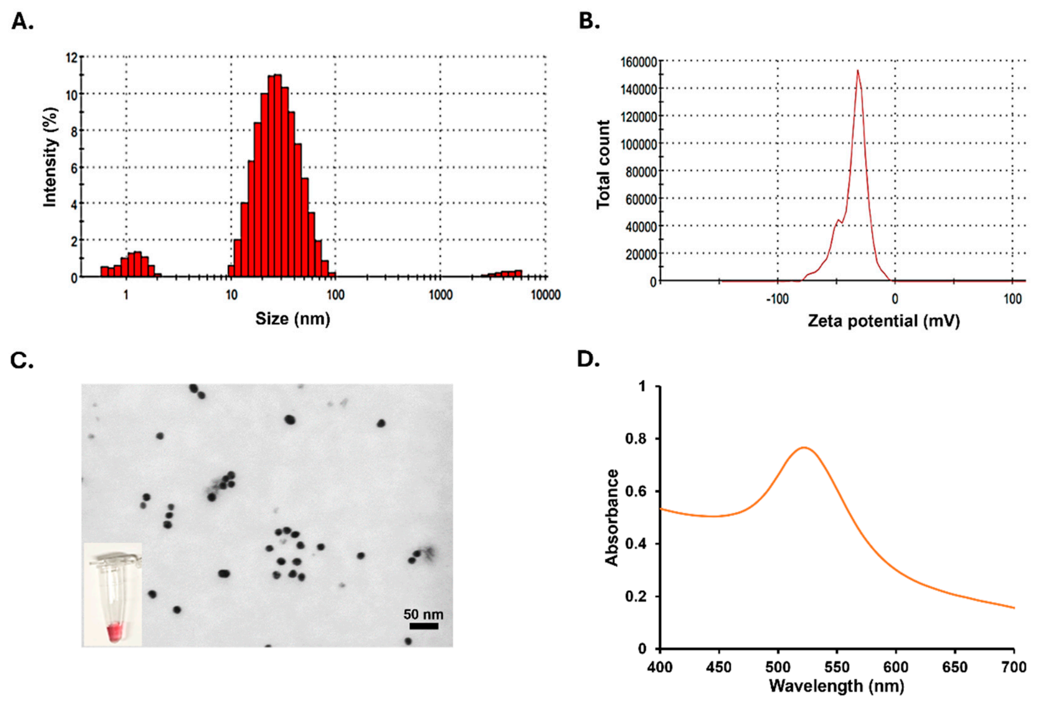

Figure S2: Gold nanoparticle (AuNP) synthesis and characterization. Characterization of the AuNPs by size distribution (A), zeta potential measurements (B), STEM image (C), and spectral analysis at the wavelength between 400 and 700 nm (D), confirming successful synthesis.

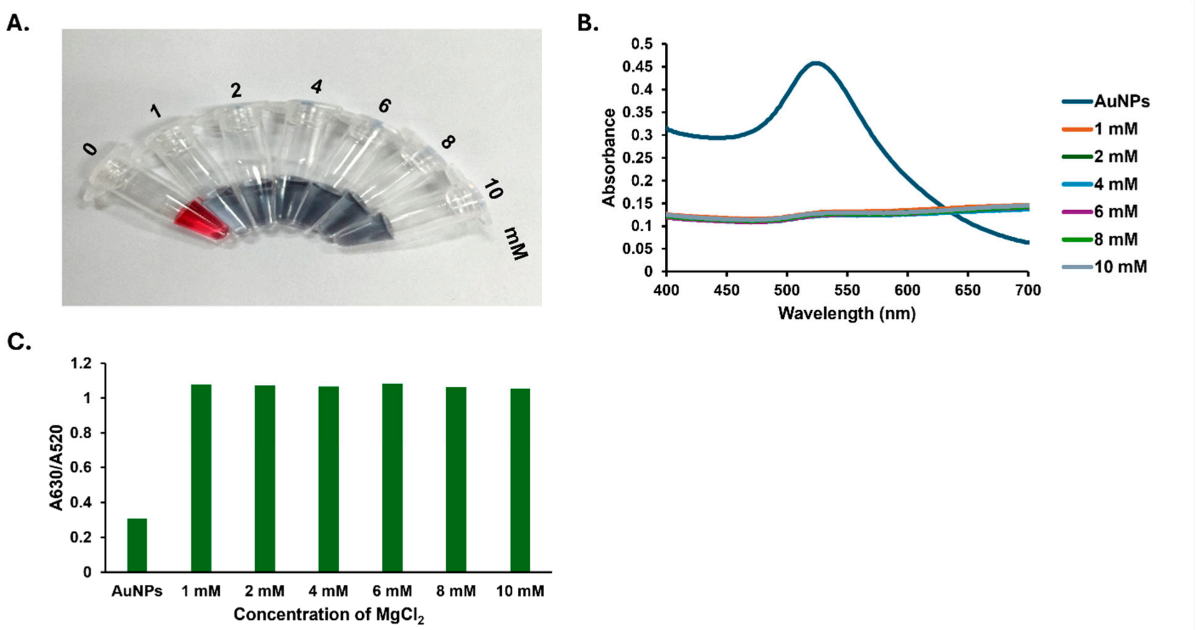

Figure S3: The effect of $MgCl_2$ on the aggregation of unmodified AuNPs. Different concentrations of $MgCl_2$ were added to unmodified AuNPs, and the color changes, absorbance spectra, and the A630/520 absorbance ratio were analyzed. (A) The aggregation of unmodified AuNPs in response to increasing $MgCl_2$ concentrations (1, 2, 4, 6, 8, 10 mM). A visible color change from reddish to purple-gray started at 1 mM $MgCl_2$ or above. (B) The UV–visible absorbance spectra of unmodified AuNPs suspensions with different $MgCl_2$ concentrations, demonstrating a redshift at 1 mM $MgCl_2$ or higher. (C) The increase in absorbance ratio (A630/520) following $MgCl_2$ addition.

Reference

1. Escudero-Abarca BI, Suh SH, Moore MD, Dwivedi HP, Jaykus LA. Selection, characterization and application of nucleic acid aptamers for the capture and detection of human norovirus strains. PLoS One. 2014;9(9):e106805.
